# SjCa8, a calcium-binding protein from *Schistosoma japonicum*, inhibits cell migration and suppresses nitric oxide release of RAW264.7 macrophages

**DOI:** 10.1186/s13071-015-1119-4

**Published:** 2015-10-07

**Authors:** Ji Liu, Tong Pan, Xu You, Yiyue Xu, Jinyi Liang, Yanin Limpanont, Xi Sun, Kamolnetr Okanurak, Huanqin Zheng, Zhongdao Wu, Zhiyue Lv

**Affiliations:** Zhongshan School of Medicine, Sun Yat-sen University, 74 2nd Zhongshan Road, Guangzhou, 510080 China; Key Laboratory for Tropical Diseases Control of Ministry of Education, Sun Yat-sen University, Guangzhou, 510080 China; Department of Social and Environmental Medicine, Faculty of Tropical Medicine, Mahidol University, Bangkok, 10400 Thailand

**Keywords:** SjCa8, *Schistosoma japonicum*, Immune evasion, Macrophage, NO, Calcium-binding protein

## Abstract

**Background:**

Schistosomiasis is considered second only to malaria as the most devastating parasitic disease in tropical countries. Schistosome cercariae invade the host by penetrating the skin and migrate though the lungs and portal circulation to their final destination in the hepatic portal system and eventually the mesenteric veins. Previous studies have shown that the cytotoxic pathways that target schistosomulum in the lung-stage involve nitric oxide (NO) produced by macrophages. By contrast, skin-stage schistosomulas can evade clearance, indicating that they might be freed from macrophage NO-mediated cytotoxicity to achieve immune evasion; however, the critical molecules and mechanisms involved remain unknown.

**Methods:**

Recombinant SjCa8 (rSjCa8), an 8-kDa calcium-binding protein that is stage-specifically expressed in cercaria and early skin-stage schistosomulas of *Schistosoma japonicum*, was incubated with mouse RAW264.7 macrophages. Effects on macrophage proliferation were determined using Cell Counting Kit-8. Next, transwell assay was carried out to further investigate the role of rSjCa8 in macrophage migration. The effects of rSjCa8 on macrophage apoptosis were evaluated using confocal microscopy and flow cytometry. Additional impacts of rSjCa8 on NO release by lipopolysaccharide (LPS)-stimulated macrophages as well as the underlying mechanisms were explored using fluorescent probe, nitric oxide signaling pathway microarray, quantitative real-time PCR, mutagenesis, and neutralizing antibody approaches.

**Results:**

rSjCa8 exhibited a striking inhibitory effect on macrophage migration, but did not markedly increase cell proliferation or apoptosis. Additionally, rSjCa8 potently inhibited NO release by LPS-stimulated macrophages in a dose- and time-dependent manner, and the inhibitory mechanism was closely associated with intracellular Ca^2+^ levels, the up-regulation of catalase expression, and the down-regulation of the expression of 47 genes, including *Myc*, *Gadd45a*, *Txnip*, *Fas*, *Sod2*, *Nos2*, and *Hmgb1*. Vaccination with rSjCa8 increased NO concentration in the challenging skin area of infected mice and reduced the number of migrated schistosomula after skin penetration by cercariae.

**Conclusions:**

Our findings indicate that SjCa8 might be a novel molecule that plays a critical role in immune evasion by *S. japonicum* cercaria during the process of skin penetration. The inhibitory impacts of rSjCa8 on macrophage migration and [Ca^2+^]_i_-dependent NO release suggest it might represent a novel vaccine candidate and chemotherapeutic target for the prevention and treatment of schistosomiasis.

**Electronic supplementary material:**

The online version of this article (doi:10.1186/s13071-015-1119-4) contains supplementary material, which is available to authorized users.

## Background

Schistosomiasis, a parasitic zoonosis caused by schistosomes that parasitize the portal and mesenteric veins of the host, remains the second most common epidemic tropical disease worldwide, as this parasite infects 200 million individuals in 73 countries in Asia (including China), Africa, and Latin America, and is responsible for ~100,000 deaths annually [1, 2]. Schistosome is a multicellular organism with a complex life cycle, in which a sophisticated immunoevasion system impedes the development of a protective immune response [3, 4]. Because praziquantel represents the only effective drug against schistosomiasis, there is a great risk of the development of drug resistance [5], which represents a severe challenge in the field of schistosomiasis prevention and eradication.

The cercaria stage is a transient free stage in the *S. japonicum* life cycle, and cercaria invasion initiates the process of infection in its definitive host. Although cercariae are relatively simple and short-lived organisms, it is well recognized that the ultraviolet-ray (UV) attenuated cercariae can induce high and stable protection against *Schistosoma mansoni* challenge in many animal models [6, 7]. Furthermore, in contrast with most lung-stage schistosomula that are eliminated by macrophage NO-mediated cytotoxicity [8, 9], skin-stage schistosomulas can evade clearance by immune cells by penetrating the host’s skin surface, indicating that a specific schistosome-derived molecule might exist that facilitates immunoevasion [10].

Our previous studies have shown that SjCa8, an 8-kDa calcium-binding protein derived from *S. japonicum*, is specifically expressed in cercariae and skin-stage schistosomula, but is silenced in eggs, lung-stage schistosomula, and adult worms [11]. Additionally, previous studies have localized SjCa8 to the cercarial head gland, penetration gland, secretions, and tegument (where cercariae directly contact and interact with host cells) [11], which suggest that stage-specific expression of SjCa8 could play a role in the skin penetration process by cercariae, although the mechanisms involved remain unclear. Therefore, in this study, we characterized the effects of rSjCa8 on macrophage proliferation, apoptosis, migration, and NO release.

## Methods

### Preparation of recombinant proteins

Purified recombinant SjCa8 (rSjCa8) and Sj13 (rSj13, control protein) were prepared as described previously [12] and subsequent endotoxin removal from purified proteins was performed using Detoxi-Gel Endotoxin Removal Columns (Thermo Fisher Scientific, Waltham, MA, USA), according to the manufacturer’s instructions [13].

To prepare recombinant mutant SjCa8 (rmuSjCa8), full-length (210 bp) *muSjCa8* DNA with site-specific mutations of glutamate (E, positions 26 and 62) in the Ca^2+^-binding loops, which are thought to be key residues for calcium binding [11, 14], to glutamine (Q), was synthesized by Takara Bio Inc. (Dalian, China). The mutant gene was subsequently cloned, expressed, purified, and subjected to endotoxin removal following established procedures [11, 13].

### Cell culture

The mouse RAW264.7 macrophage-like cell line was obtained from the American Type Tissue Culture Collection (Rockville, MD, USA), cultured in Dulbecco’s Modified Essential Medium (DMEM, Gibco® Life Technologies, Grand Island, NY, USA) supplemented with 10 % heat-inactivated fetal bovine serum (FBS, Gibco® Life Technologies) and penicillin (100 U/ml) plus streptomycin (100 μg/ml), and incubated at 37 °C and 5 % CO_2_. Cells were seeded in a T25 flask at 5 × 10^5^ cells/ml 1 day prior to inoculation.

### Cell proliferation assay

RAW264.7 cells were seeded in 96-well culture plates 1 day prior to treatment at a density of 2 × 10^3^ cells/well in 100 μL medium, and then were treated with either phosphate buffer saline (PBS), 20 μg/ml rSj13, 5 μg/ml concanavalin-A (ConA, positive control), or rSjCa8 at different concentrations (1 and 20 μg/ml) for either 24 or 48 h. Cell viability was assessed using Cell Counting Kit-8 (CCK-8, Beyotime Biotechnology, Haimen, China) as described previously [15]. Absorbance at 450 nm, for positive values indicate cellular proliferation, was measured using a reference wavelength of 595 nm with an automatic Microplate Absorbance Reader (Tecan, Austria).

### Apoptosis assay

To detect apoptosis in RAW264.7 cells after rSjCa8 treatment, we assessed the surface phosphatidylserine (PS) expression levels, which increase during apoptosis. Exposure of the phospholipid PS to the external leaflet of the plasma membrane occurs early in the process of apoptosis and can be measured by Annexin-V-FITC staining. Moreover, dead cells that reach late stages of apoptosis can be quantified by positive PI staining. To quantify apoptosis, an early Apoptosis Detection Kit (Beyotime Biotechnology) was performed. RAW264.7 cells (10^5^ cells/well) were grown in culture medium in the presence or absence of rSjCa8 (1 or 20 μg/ml), rSj13 (20 μg/ml), or 1:2000 (V/V) Apoptosis Inducer (positive control, Beyotime Biotechnology) for 24 or 48 h. Then, cells were harvested and pelleted by centrifugation. After washing with PBS and resuspension in binding buffer (10 mM HEPES/NaOH, 140 mM NaCl, 2.5 mM CaCl_2_, pH 7.4) containing 1:100 (V/V) propidium iodide (PI) and Annexin V-FITC at room temperature for 30 min in the dark, cells were analyzed using either a Beckman CytoFLEX Flow Cytometer (Beckman Coulter, Brea, CA, USA), or observed using a 40× lens under an Olympus FV 500-IX81 laser-scanning confocal microscope (Olympus Microscopy, Hamburg, Germany) with an excitation wavelength of 490 nm and emission wavelengths of 520 nm for Annexin-V-FITC and 650 nm for PI. The apoptosis rate for each group was calculated according to the following formula: apoptosis rate (%) = (number of apoptotic cells in a microscopic field/total number of cells in a microscopic field) × 100.

### Transwell migration assay

*In vitro* migration assays were performed using transwell chambers with a 10-μm thin transparent polycarbonate membrane and an 8-micron polyester filter (Corning Inc. Corning, NY, USA). RAW264.7 cells (7.5 × 10^3^) that were pretreated for 1 h with 20 μg/ml rSj13 or 0, 1, or 20 μg/ml rSjCa8 were added to the upper compartment where 1 % FBS DMEM was added, whereas DMEM containing 10 % FBS was added to the lower compartment. After 30 h incubation, the membrane between the two compartments was removed, fixed in methanol for 10 min, and stained with Giemsa reagent. Cells on the lower surface of the filter were photographed under an Olympus BX51WI microscope (Olympus Microscopy) and counted. Five random views were photographed and quantified.

### Measurement of intracellular nitric oxide production

Intracellular NO release in individual RAW264.7 cells was determined using a fluorescent dye for NO, 4-amino-5-methylamino-2ʹ,7ʹ-difluorofluorescein diacetate (DAF-FM/DA, Beyotime Biotechnology) following a previously described protocol [16]. To assess the effect of rSjCa8 on NO production by macrophages, RAW264.7 cells were pre-stimulated with 1 μg/ml LPS for 24 h at 37 °C, and then were treated for 30 min with various doses of rSjCa8 (0, 1, and 20 μg/ml) or control protein rSj13 (20 μg/ml). To analyze the dose- and time-dependent inhibitory activity of rSjCa8 on NO production by macrophages, LPS-stimulated RAW264.7 cells were incubated with 20 μg/ml rSjCa8 for 0, 1, 10, 20, or 30 min. Cells from all groups were loaded with 5 μM DAF-FM/DA in the dark for 30 min at 37 °C, and then were rinsed three times. Thereafter, the fluorescence intensities of cells were measured using an Olympus FV 500-IX81 laser-scanning confocal microscope (Olympus, Microscopy) with excitation and emission wavelengths of 490 and 510 nm, respectively. Furthermore, dynamic changes of fluorescence intensities of NO produced by LPS-stimulated macrophages after treatment with increasing concentrations of rSjCa8 (5, 10, 20, 40, 60 and 80 μg/ml) were recorded in time-series mode at 1.7 s intervals under a confocal microscope.

### Detection of intercellular Ca^2+^ levels

Previous studies have shown a role for thapsigargin as an endoplasmic reticulum (ER) stressor, as well as a Ca^2+^-pump (ATPase) inhibitor that acts on the sarcoplasmic reticulum (SR) membrane [17]. In the ER stress response, apoptosis is induced by thapsigargin in a manner dependent upon Ca^2+^ outflow from the SR along with cytoplasmic calcium overload. EDTA functions as a chelator bound to Ca^2+^ to reduce intercellular calcium concentrations. To test for correlations between NO production and intracellular calcium concentrations ([Ca^2+^]_i_) in macrophages, we simultaneously detected intracellular NO production and [Ca^2+^]_i_ of individual RAW264.7 cells after various treatments. Cells pretreated with LPS (1 μg/ml) for 24 h were divided into 6 groups: controls treated with PBS (group I), a group treated with 20 μg/ml rSjCa8 for 30 min (group II), an internal control group treated with 20 μg/ml rSj13 for 30 min (group III), a group treated with 20 μg/ml rSjCa8 + 10 mM ethylenediaminetetra-acetic acid (EDTA, a chelator of Ca^2+^) for 30 min (group IV), a group treated with 20 μg/ml rSjCa8 + 30 μM thapsigargin (an inhibitor of sarco-endoplasmic reticulum Ca^2+^-ATPases) for 30 min (group V), and a group treated with 20 μg/ml rmuSjCa8 (group VI).

Finally, cells were loaded with 2 μM Rhod-2 AM (Dojindo Kumamoto, Kumamoto, Japan; a red fluorescent probe for intercellular Ca^2+^) and 5 μM DAF-FM/DA (a NO indicator) in the dark for 30 min at 37 °C. After incubation, cells were imaged using an Olympus FV 500-IX81 laser-scanning confocal microscope (Olympus Microscopy). The fluorescence of Rhod-2 AM and DAF-FM/DA were excited at 577 nm and 490 nm, and emitted at 581 nm and 510 nm, respectively.

### NO PCR microarray chip

RAW264.7 cells were cultured, stimulated with 1 μg/ml LPS for 24 h, and treated with either PBS or rSjCa8 (20 μg/ml) for 30 min as described above. Total RNA from cells in both groups was prepared using TRIzol® reagent (Invitrogen Life Technologies, Carlsbad, CA, USA) and the Mouse Nitric Oxide Signaling Pathway RT^2^ Profiler™ PCR Array (SABiosciences/QIAGEN Company, Fredrick, MD, USA) was performed according to the manufacturer’s protocol by KangChen Bio-Tech Inc. (Shanghai, China). In the statistical analyses, gene expression differences were considered significant if they showed an expression fold-change ≥3 between two groups with a *p*-value <0.05.

### Real-time quantitative PCR

A subset of genes exhibiting different expression patterns was selected for further validation using qRT-PCR. Reactions were carried out in technical triplicates using a LightCycler®480 System (Roche, Switzerland) with universal cycling conditions, as described in our previous study [13]. Specific primers for target genes and the reference gene, *18S* rRNA, are listed in Table 1, and the relative expression levels of each selected gene were quantified by normalization to the corresponding normalized value of the *18S* rRNA control (fold-change = 2^−ΔΔCT^) using the software provided with the instrument.

**Table 1:** Primers used for quantitative RT- PCR

Gene symbol	Forward primer	Reverse primer	Ref.
*Cat*	CCTCGTTCAGGATGTGGTTT	GGCATCCCTGATGAAGAAAA	[49]
*Fas*	GCAGACATGCTGTGGATCTGG	TCACAGCCAGGAGAATCGCAG	[50]
*Gadd45a*	CTGCCTCCTGGTCACGAA	TTGCCTCTGCTCTCTTCACA	[51]
*Hmgb1*	CCATTGGTGATGTTGCAAAG	CTTTTTCGCTGCATCAGGTT	[52]
*Myc*	GAGGCGAACACACAACGTCTT	CACGCAGGGCAAAAAAGC	[53]
*NOS2*	AACCCCTTGTGCTGTTCTCAGCC	GTGGACGGGTCGATGTCACATGC	[54]
*Sod2*	ATGTTACAACTCAGGTCGCTCTTC	TGATAGCCTCCAGCAACTCTCC	[55]
*Txnip*	CAAGTTCGGCTTTGAGCTTC	GCCATTGGCAAGGTAAGTGT	[56]
*18S*	GTCTGTGATGCCCTTAGA	AGCTTATGACCCGCACTTAC	[57]

### Worm recovery and tissue sampling

To evaluate the effects of rSjCa8 on NO production and host skin penetration by *S. japonicum* larvae *in vivo*, a modified immune challenge experiment in mice was performed as described in our previous study [11]. Briefly, 40 mice were randomly divided into four groups of 10 mice each. The immune challenge group (Freund’s adjuvant + rSjCa8/infected group), adjuvant-treated group (Freund’s adjuvant/infected group), and challenge group (infected group) were treated as described previously [11]. The immune-challenge group of mice was injected subcutaneously with 20 μg rSjCa8 plus complete Freund’s adjuvant (Sigma) and were boosted twice with the same amount of antigen with incomplete Freund’s adjuvant (Sigma) at 2-week intervals. The adjuvant-treated group of mice was subjected to the same immunization schedule as the immune-challenge group, but PBS replaced rSjCa8. Fourteen days after the final boost, mice in these three groups were challenged percutaneously with 50 ± 2 cercariae for 20 min using the cover glass method. Another group of 10 mice (uninfected group) was subjected to the same immunization schedule as the adjuvant group, but PBS was used in place of adjuvant and without a subsequent challenge infection with cercariae.

At 6 h post-challenge infection, all vaccinated and control mice were euthanized and the shaved skin areas that were exposed to cercariae were quickly removed. The skin tissues from half of each group of mice were cut into pieces and cultured for 24 h at 37 °C, 5 % CO_2_ in RPMI 1640 medium containing antibiotics (100 U/ml penicillin and 100 μg/ml streptomycin) and 10 % fetal calf serum (Invitrogen). In these cultured skin tissues, the effects of rSjCa8 on penetration and migration by cercariae were evaluated based on the number of penetrated larvae, as described previously [18]. The skin tissues from the other half of each group were cut into pieces and lysed with Lysis Buffer (Thermo Scientific/Pierce; 1 ml/g skin), and skin homogenates were prepared using a TissueLyser II (Qiagen) and centrifuged at 10,000 g for 5 min at 4 °C to collect supernatants. The Total Nitric Oxide Assay kit (Beyotime Biotechnology) was used for NO detection following the manufacturer’s protocol and NO levels in the skin homogenate supernatants were determined by measuring nitrites with Griess reagent [19]. Absorbance was read at 540 nm with an automatic Microplate Absorbance Reader (Tecan, Austria).

### Statistical analysis

Each experiment was repeated three times and data were represented as means ± standard derivation (SD). The statistical significance of differences between groups was determined using a one-way analysis of variance (ANOVA) followed by the Tukey–Kramer test using GraphPad Prism version 5.0 (GraphPad Software Inc., La Jolla, CA, USA); *P* < 0.05 was used as a threshold for statistically significant differences.

### Ethical Statement

This study was carried out in strict accordance with the recommendations in the Guide for the Care and Use of Laboratory Animals of Sun Yat-sen University. The protocol was approved by the Committee on the Ethics of Animal Experiments of Zhongshan School of Medicine, Sun Yat-sen University (Permit Number: 2010–0326). All surgery was performed under sodium pentobarbital anesthesia, and all efforts were made to minimize suffering.

## Results

### rSjCa8 does not affect macrophage proliferation

**Fig. 1:**
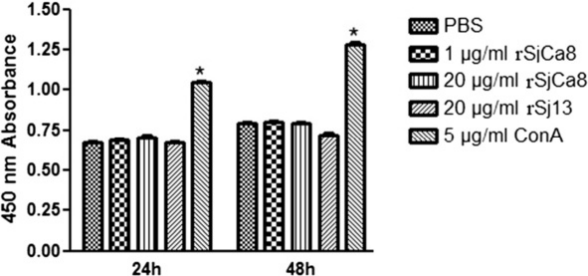
Effects of rSjCa8 on the proliferation of RAW264.7 cells. RAW264.7 cells were treated or not with ConA (5 μg/ml), rSj13 (20 μg/ml), or various concentrations of rSjCa8 (1 or 20 μg/ml) for 24 or 48 h, respectively. Cell viability was assessed in triplicate using the CCK-8 method according to the manufacturer’s protocol. * *P*<0.05, compared with the PBS group

To assess the effects of rSjCa8 on the growth of RAW264.7 cells, cell viability was evaluated using the CCK-8 method. Notably, the ConA (positive control) used at test concentration significantly induced cell proliferation compared with that observed in the PBS group, whereas neither rSjCa8 (1, 5, or 20 μg/ml) nor rSj13 altered the proliferation of RAW264.7 cells (Fig. 1).

### rSjCa8 does not induce macrophage apoptosis

**Fig. 2:**
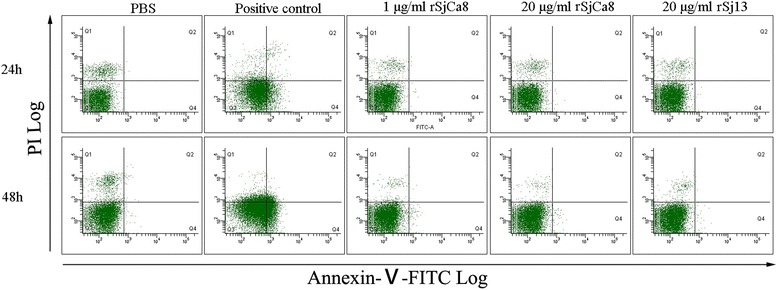
Effects of rSjCa8 on the apoptosis of RAW264.7 cells. RAW264.7 cells were treated with PBS, an apoptosis inducer (positive control), 20 μg/ml rSj13, or 1 and 20 μg/ml rSjCa8 for 24 or 48 h. Annexin-V-FITC-PI staining allows live, apoptotic, and dead cells to be identified using a flow cytometer. The x-axis indicates the Annexin V-positive cells, and the y-axis displays PI-positive cells. This experiment was performed in triplicate

As shown in Fig. 2, an apoptosis inducer dramatically increased the apoptosis of RAW264.7 cells compared with the PBS and rSj13 control group, with proportions of 35.2 and 46.1 % Annexin V-positive cells after 24 and 48 h of treatment, respectively, whereas no obvious changes in the proportion of Annexin V-positive cells after stimulation for 24 or 48 h by 1 μg/ml (2.35 and 4.16 %, respectively) or 20 μg/ml (1.98 and 3.77 %, respectively) rSjCa8 were observed (see the lower right quadrant of the dot plot), indicating a negligible effect of rSjCa8 on macrophage apoptosis.

**Fig. 3:**
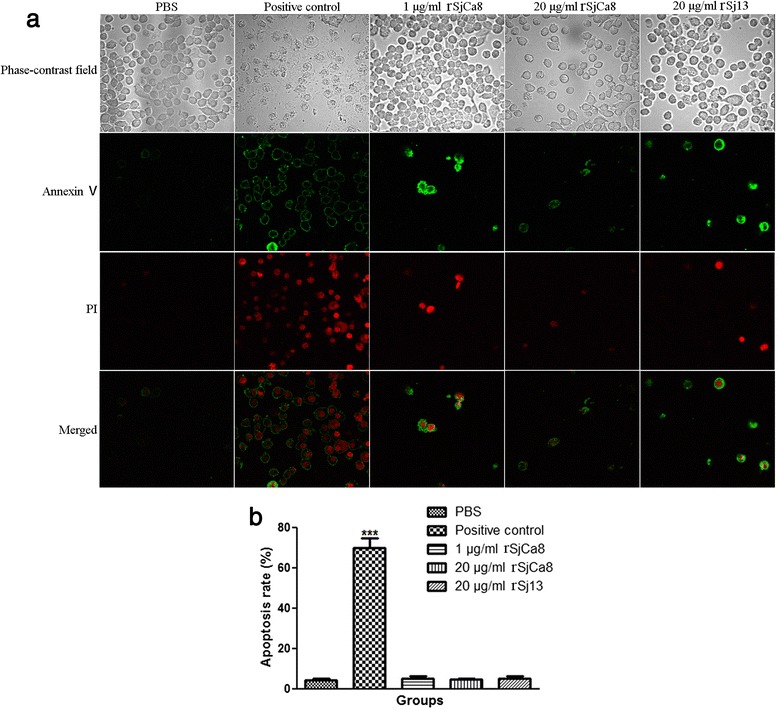
Confirmation of the effects of rSjCa8 on RAW264.7 cell apoptosis by confocal microscopy. Cells were exposed to Annexin-V-FITC and PI for 30 min prior to analysis. The fluorescence of apoptotic cells was detected under a confocal microscope (**a**) and the apoptosis rate was calculated (**b**). The graph shows the means of three independent experiments that were performed in triplicate

To confirm this observation, apoptosis imaging by confocal microscopy was performed. Similar to our flow cytometry data, there was a significant 16.14-fold increase (*P* < 0.001) in apoptotic cells, which were labeled by Annexin-V-FITC and PI, in the positive control group compared with that observed in the PBS control group. However, differences between the PBS group, 1 μg/ml rSjCa8 group, 20 μg/ml rSjCa8 group, and 20 μg/ml rSj13 group were not significant (*P* > 0.05; Fig. 3). Thus, we concluded that rSjCa8 treatment did not significantly affect macrophage apoptosis.

### rSjCa8 inhibits macrophage migration

**Fig. 4:**
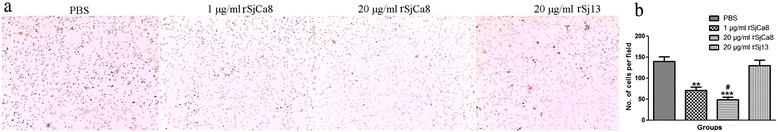
Impaired migration of RAW264.7 cells caused by rSjCa8 in a transwell chamber assay. Filters were stained by Giemsa reagent (**a**) and the cells that passed through the filter were counted (**b**). The capacity of RAW264.7 cells to migrate through transwell filters was significantly inhibited by treatment of cells with 1 μg/ml rSjCa8 (*P* < 0.01) or 20 μg/ml rSjCa8 (*P* < 0.001), but not rSj13, compared with the PBS group. The means ± SD of three independent experiments are shown; **P < 0.01 and ***P < 0.001, compared with the PBS group; #*P* < 0.05, compared with the 1 μg/ml SjCa8 group

Down-regulation of host macrophage chemotaxis facilitates immune evasion by cercariae of *S. japonicum* when they penetrate the skin. We explored the potential effect of rSjCa8 on macrophage migration using a transwell system. Our results revealed that rSjCa8 treatment dose-dependently and markedly reduced macrophage migration compared with the control group (1 μg/ml rSjCa8 group vs. PBS group, *P* < 0.01; 20 μg/ml rSjCa8 group vs. PBS group, *P* < 0.001; Fig. 4). Therefore, rSjCa8 might play a vital role in limiting macrophage chemotaxis, especially when *S. japonicum* cercariae penetrate the skin of the hosts, thereby down regulating the host immune response and facilitating infection.

### rSjCa8 inhibits LPS-induced NO release by RAW264.7 cells

**Fig. 5:**
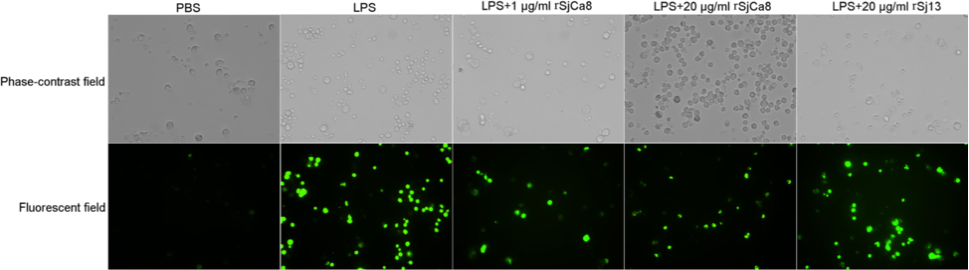
Inhibition of NO release by LPS-stimulated RAW264.7 cells treated with rSjCa8. RAW264.7 cells were cultured in medium containing 1 μg/ml LPS for 24 h, and were subsequently exposed for another 30 min to PBS, rSjCa8 (1 or 20 μg/ml), or rSj13 (20 μg/ml). NO generation by the cells that were loaded with the NO-specific fluorescent probe DAF-AM was detected as intracellular green fluorescence signal by laser-scanning confocal microscopy

**Fig. 6:**
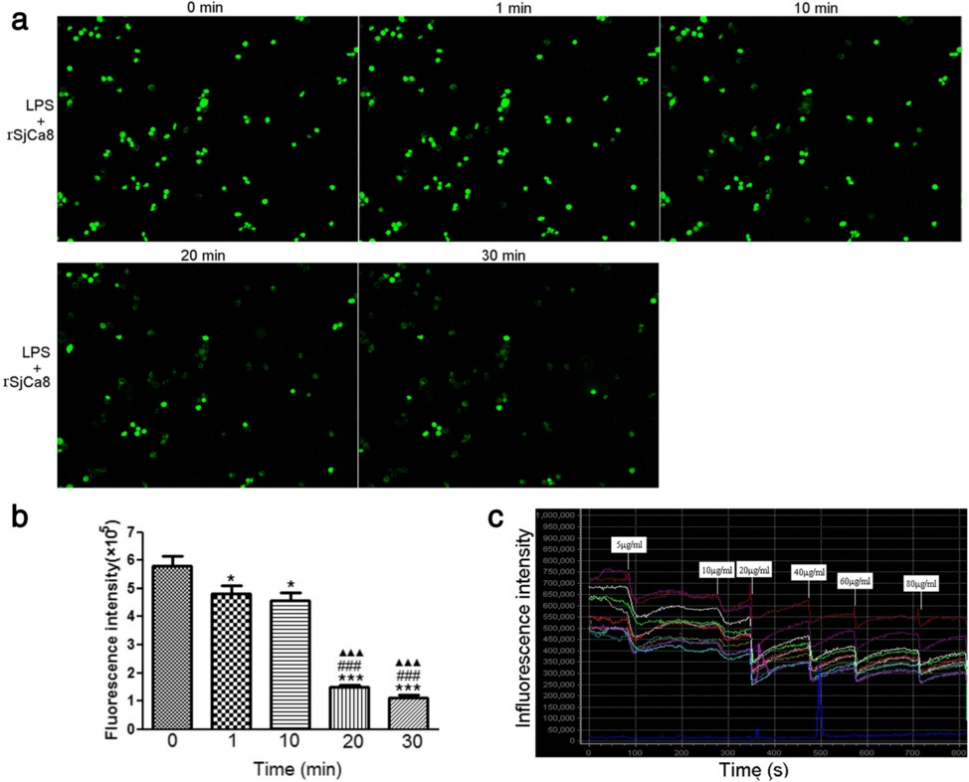
Time- and dosage-dependent inhibitory effects of rSjCa8 on NO production by macrophages stimulated with LPS. Quantification of the NO signal intensity in response to treatment with 20 μg/ml rSjCa8 for 1, 10, 20, or 30 min (**a**). Fluorescence of the entire area of RAW264.7 cells that was captured in the microscope field of view was quantified by measuring the fluorescence intensity (**b**). Dynamic changes in NO release in LPS-stimulated macrophages exposed to increasing levels (0–80 μg/ml) of rSjCa8 observed by confocal microscopy were recorded (**c**). Data represent means ± SD of three experiments; ∗, *P* < 0.05; ∗∗∗, *P* < 0.001 compared with the group not treated with rSjCa8; ###, *P* < 0.001 compared with the group treated with rSjCa8 for 1 min; ▲▲▲, *P* < 0.001 compared with the group treated with rSjCa8 for 10 min

Figure 5 shows NO release in cultured LPS-stimulated RAW264.7 macrophages exposed to rSjCa8 and the fluorescence intensity of NO increased in macrophages 24 h after stimulation with 1 μg/ml LPS. Surprisingly, NO generation in RAW264.7 cells treated with LPS was significantly suppressed by rSjCa8 treatment in a concentration- and time-dependent manner, as indicated by the reduced intracellular fluorescent signals of the NO probes (Figs. 5 and 6). This finding is consistent with the changes in NO real-time fluorescence that we monitored using laser-scanning confocal microscopy (Fig. 6c), which quantitatively show diminished fluorescent intensity when the dosage of rSjCa8 was increased (0–20 μg/ml). These findings suggest that the disruption of NO generation in response to rSjCa8 occurs in macrophages.

### Suppression of NO release by rSjCa8 in RAW264.7 cells is correlated with changes in intracellular calcium levels

**Fig. 7:**
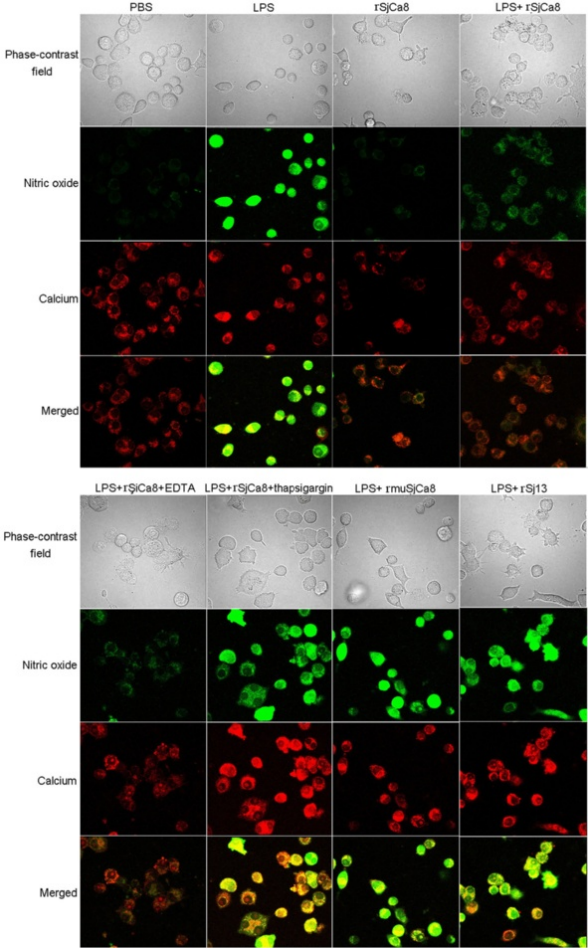
Correlations between intracellular calcium levels and reduced NO generation by rSjCa8. After incubation with or without 1 μg/ml LPS, RAW264.7 macrophages were treated with 20 μg/ml rSjCa8 plus different reagents that can alter intercellular calcium level, as described in the methods section. Cells were loaded with the NO probe DAF-AM (green) and the calcium ion probe Rhod-2 AM (red). Green and red fluorescent intensities were detected simultaneously by laser-scanning capture microscopy (600×)

**Fig. 8:**
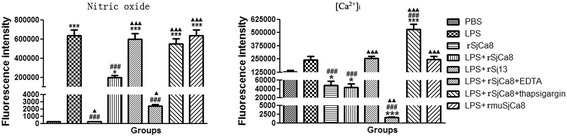
Quantification of NO and calcium fluorescence in macrophages exposed to various *in vitro* stimuli. Significant differences between means are indicated; *P < 0.05, ***P < 0.001 compared with untreated cells; ###*P* < 0.001 compared with LPS-stimulated cells; ▲ < 0.05, ▲▲ *P* < 0.01, ▲▲▲*P* <0.001 compared with LPS-stimulated cells exposed to rSjCa8

To investigate correlations between cytoplasmic Ca^2+^ levels and the suppressive effects of rSjCa8 on NO release, either chelate (EDTA), an agonist (thapsigargin) of calcium, or a recombinant mutant SjCa8 protein (rmuSjCa8) were applied to either increase or decrease levels of intracellular Ca^2+^ in macrophages. The data shown in Figs. 7 and 8 indicate that both cytoplasmic Ca^2+^ levels and NO production after LPS treatment increased compared with the PBS controls. Co-treatment with rSjCa8 and EDTA impaired NO generation and caused the elevation of Ca^2+^ levels, whereas NO production and intracellular Ca^2+^ levels were markedly enhanced in test groups treated with either thapsigargin or rmuSjCa8. Therefore, our findings indicate that NO production by macrophages stimulated with LPS may depend upon increases in cytosolic Ca^2+^ levels. Moreover, the suppressive effects of rSjCa8 on NO release by LPS-stimulated macrophages might be a consequence of reduced intracellular Ca^2+^ concentrations. Moreover, Glutamate residues at positions 26 and 62 in the Ca^2+^-binding loops might be critical for the Ca^2+^ signaling inhibitory bioactivity of rSjCa8 that affects NO production.

### Transcriptional profiles of genes involved in the NO signaling pathway are markedly altered in LPS-stimulated macrophages treated with rSjCa8

To elucidate the mechanism of rSjCa8-mediated inhibitory effects on NO production in LPS-stimulated macrophages, we attempted to identify genes with altered expression levels after treatment with rSjCa8. We focused on the nitric oxide pathway by quantitative real-time PCR microarray analysis to catalogue the expression of 84 genes, and identified 47 genes that were downregulated more than 3-fold, whereas 1 gene showed a greater than 3-fold up-regulation (Table 2 and Additional file 1: Table S1). Notably, *Myelocytomatosis oncogene* (*Myc*, a transcription factor), *Growth arrest and DNA-damage-inducible 45 alpha* (*Gadd45a*, a gene that responds to environmental stresses), *Thioredoxin interacting protein* (*Txnip*, an oxidative stress mediator), *TNF receptor superfamily member 6* (*Fas*, an inducer of apoptosis), *Superoxide dismutase 2* (*Sod2*, an anti-oxidant gene), *High mobility group box 1* (*Hmgb1*, a cytokine mediator of inflammation), and *Nitric oxide synthase 2* (*Nos2*, an enzyme that catalyzes the production of NO) were down-regulated by 1868.05-, 367.26-, 325.89-, 279.80-, 232.28-, 41.98-, and 15.64-folds, respectively, whereas the expression of the anti-oxidative stress gene *catalase* (*Cat*) was up-regulated by 1012.32-fold (Table 2).

**Table 2:** General information of differentially expressed genes from LPS + rSjCa8 treated macrophages compared to LPS treated macrophages

Well	RefSeq	Symbol	Description	T-test	Fold Difference
				*P* value	(LPS + rSjCa8)/LPS
A02	NM_028717	Als2	Amyotrophic lateral sclerosis 2 (juvenile) homolog (human)	3.38E-04	−4.14
A04	NM_007527	Bax	Bcl2-associated X protein	6.11E-08	10.79
A05	NM_009743	Bcl2l1	Bcl2-like 1	4.58E-06	−3.43
A08	NM_009795	Capns1	Calpain, small subunit 1	1.58E-03	−3.57
A09	NM_009804	Cat	Catalase	4.22E-07	1012.32
B01	NM_016892	Ccs	Copper chaperone for superoxide dismutase	2.32E-08	−13.69
B03	NM_007669	Cdkn1a	Cyclin-dependent kinase inhibitor 1A (P21)	1.47E-04	−7.75
B05	NM_007806	Cyba	Cytochrome b-245, alpha polypeptide	2.05E-04	−7.35
B08	NM_007864	Dlg4	Discs, large homolog 4 (Drosophila)	8.65E-05	−9.50
C01	NM_007987	Fas	Fas (TNF receptor superfamily member 6)	1.30E-04	−279.80
C02	NM_010234	Fos	FBJ osteosarcoma oncogene	3.73E-05	−14.66
C04	NM_007836	Gadd45a	Growth arrest and DNA-damage-inducible 45 alpha	2.80E-08	−367.26
C05	NM_008160	Gpx1	Glutathione peroxidase 1	1.12E-04	−5.45
C07	NM_008161	Gpx3	Glutathione peroxidase 3	2.58E-05	−23.15
D01	NM_010439	Hmgb1	High mobility group box 1	2.78E-07	−41.98
D02	NM_008281	Hpn	Hepsin	4.79E-04	−3.87
D03	NM_010497	Idh1	Isocitrate dehydrogenase 1 (NADP+), soluble	3.20E-06	−8.61
D04	NM_008326	Irgm1	Immunity-related GTPase family M member 1	2.26E-04	−8.25
D05	NM_010786	Mdm2	Transformed mouse 3 T3 cell double minute 2	1.09E-05	−14.94
D07	NM_010849	Myc	Myelocytomatosis oncogene	1.18E-09	−1868.05
D08	NM_010877	Ncf2	Neutrophil cytosolic factor 2	1.32E-04	−3.86
D09	NM_008682	Nedd1	Neural precursor cell expressed, developmentally down-regulated gene 1	2.38E-06	−18.38
D12	NM_010927	Nos2	Nitric oxide synthase 2, inducible	1.46E-06	−15.64
E02	NM_172203	Nox1	NADPH oxidase 1	1.46E-06	−3.30
E07	NM_011063	Pea15a	Phosphoprotein enriched in astrocytes 15A	3.44E-05	−11.22
E08	NM_133819	Ppp1r15b	Protein phosphatase 1, regulatory (inhibitor) subunit 15b	5.35E-08	−7.67
E09	NM_008913	Ppp3ca	Protein phosphatase 3, catalytic subunit, alpha isoform	3.61E-07	−24.03
E10	NM_011034	Prdx1	Peroxiredoxin 1	3.28E-04	−4.18
E11	NM_011563	Prdx2	Peroxiredoxin 2	1.30E-06	−12.04
E12	NM_007453	Prdx6	Peroxiredoxin 6	3.91E-04	−3.19
F02	NM_011170	Prnp	Prion protein	5.97E-05	−12.71
F06	NM_009029	Rb1	Retinoblastoma 1	2.40E-05	−16.42
F07	NM_058214	Recql4	RecQ protein-like 4	4.72E-07	−11.70
F09	NM_009101	Rras	Harvey rat sarcoma oncogene, subgroup R	4.22E-07	−16.76
F10	NM_009127	Scd1	Stearoyl-Coenzyme A desaturase 1	1.01E-07	−35.25
F11	NM_009128	Scd2	Stearoyl-Coenzyme A desaturase 2	7.52E-05	−6.71
F12	NM_024450	Scd3	Stearoyl-coenzyme A desaturase 3	3.54E-04	−7.59
G02	NM_011434	Sod1	Superoxide dismutase 1, soluble	1.78E-03	−4.30
G03	NM_013671	Sod2	Superoxide dismutase 2, mitochondrial	5.07E-08	−232.28
G06	NM_011640	Trp53	Transformation related protein 53	1.09E-04	−9.62
G07	NM_023719	Txnip	Thioredoxin interacting protein	7.36E-09	−325.89
G08	NM_013711	Txnrd2	Thioredoxin reductase 2	6.01E-08	−37.67
G09	NM_019639	Ubc	Ubiquitin C	1.28E-04	−4.20
G11	NM_026119	Med4	Mediator of RNA polymerase II transcription, subunit 4 homolog (yeast)	2.78E-06	−3.39
H01	NM_010368	Gusb	Glucuronidase, beta	4.77E-02	−5.90
H02	NM_013556	Hprt1	Hypoxanthine guanine phosphoribosyl transferase 1	1.60E-05	−23.66
H04	NM_008084	Gapdh	Glyceraldehyde-3-phosphate dehydrogenase	1.60E-04	−6.13
H05	NM_007393	Actb	Actin, beta	3.51E-03	−8.43

*-* genes that were downregulated

### Validation of differentially expressed genes detected by microarray analyses

**Fig. 9:**
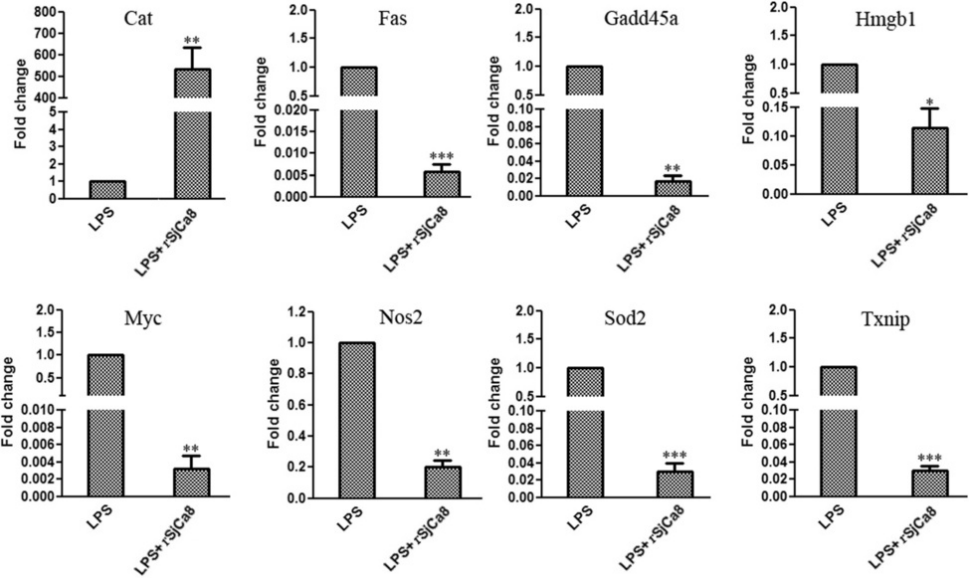
Measurements of mRNA levels of the most differentially expressed genes observed in microarrays. The eight most differentially expressed genes (*Cat*, *Fas*, *Gadd45a*, *Hmgb1*, *Myc*, *Nos2*, *Sod2*, and *Txnip*) were selected to determine mRNA expression levels by quantitative real-time PCR using specific primers. Values shown represent means ± SD; ∗, *P* <0.05; ∗∗, *P* <0.01; and ∗∗∗, *P* <0.001 compared with the LPS-stimulated group

Changes in the expression levels of the most significantly differentially expressed genes identified by the microarray analyses were validated using quantitative RT-PCR to measure individual transcript levels. As shown in Fig. 9, our RT-PCR data confirmed the significant downregulation of *Myc* (314.47-fold reduction), *Gadd45a* (58.14-fold reduction), *Txnip* (33.50-fold reduction), *Fas* (173.91-fold reduction), *Sod2* (34.22-fold reduction), *Hmgb1* (8.76-fold reduction), and *Nos2* (5.01-fold reduction), and the upregulation of *Cat* (534.67-fold increase) in the LPS-stimulated macrophages after exposure to 20 μg/ml rSjCa8 for 30 min.

### Vaccination of rSjCa8 increase NO concentration in the challenging skin area and reduce the number of larvae that penetrate and migrate through the skin

**Fig. 10:**
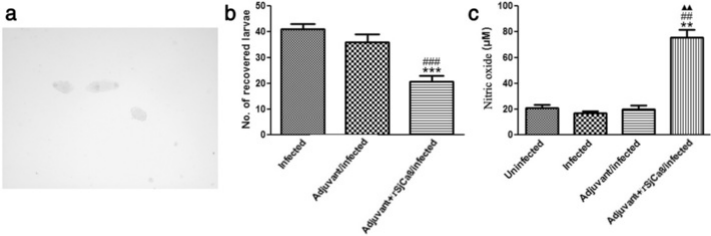
Effects of vaccination of rSjCa8 on NO production in the skin and on the number of invaded larvae. Photomicrographs (×40) of recovered schistosomula from different experimental groups as observed using a microscope (**a**). Harvested larvae of *S. japonicum* were counted (**b**), and NO concentrations in the skin homogenates were determined by absorbance at a 540-nm wavelength using a total nitric oxide assay kit; **P < 0.01, ***P < 0.001 compared with uninfected mice; ## *P* < 0.01, ###*P* < 0.001 compared with the adjuvant/infected group; ▲▲ *P* < 0.01 compared with the adjuvant + rSjCa8/infected group. Experiments were replicated three times

The inhibitory efficacy was tested by quantifying the number of skin-stage schistosomula and effects on NO release that were induced by vaccination with rSjCa8 following challenge with 50 ± 2 cercariae. Our data showed that immunization with adjuvant + rSjCa8 resulted in a significant (*P* < 0.001) reduction by 49.39 % in the number of larvae that we recovered compared with that recovered from control mice (Fig. 10b). By contrast, NO release in skin homogenates was significantly increased in infected animals after vaccination with adjuvant + rSjCa8 compared with that detected in uninfected mice, infected mice without vaccination, or infected mice with adjuvant vaccination alone (*P* < 0.01; Fig. 10c). The numbers of recovered schistosomula from infected mice versus infected mice treated with adjuvant, and the NO levels among uninfected mice, infected mice without vaccination, and infected mice with adjuvant vaccination alone were not significantly different (Fig. 10b, c).

## Discussion

Ca^2+^ and NO are two important cellular messengers, and Ca^2+^ fluxes have been recognized to account for NO production [20]. Researchers have confirmed that both Ca^2+^ fluxes and calcium-related proteins, such as regucalcin, calmodulin, and CBPs, are thought to affect NO synthesis and release by a range of somatic cells, including renal cortical cells, neurons, macrophages, and plant cells [21–25]. In the innate immune response, calcium-binding proteins act as potential sensors that are activated by pathogen-induced calcium influx. Increased cytoplasmic Ca^2+^ levels trigger the activation of various downstream protein targets that affect numerous signal transduction cascades, including the NOS (nitric oxide synthase) pathway that has been associated with increased NO generation after pathogen infection *in vivo* and *in vitro* [26]. However, the importance of calcium-binding proteins from pathogens (e.g., parasites, bacteria, or viruses) on macrophage release or functions in the host has not yet been directly established [27–29]. To date, SjCa8 is the only CBP known to be stage-specifically expressed in schistosomal cercaria and skin-stage schistosomula [11]. Our findings originally indicated that rSjCa8 could significantly inhibit macrophage migration and NO release, despite its minimal impact on macrophage proliferation and apoptosis. Furthermore, our present findings show that mutations in the EF-hand motif of rSjCa8 or administration of thapsigargin (an agonist of store-operated Ca^2+^ channels) can reverse rSjCa8-induced inhibitory effects on NO release in LPS-stimulated macrophages demonstrated that the inhibition effects might be regulated by Ca^2+^-dependent signaling. We have now made the exciting observation that rSjCa8 can both modulate intracellular Ca^2+^ levels and also inhibit macrophage migration and NO production in the cercariae-challenged host, thereby suppressing host cell-mediated killing or elimination effects on a pathogen.

Recent work indicates that LPS, a ubiquitous component of gram-negative bacteria, can elicit innate immune responses in both animals and plants by functioning as a pathogen-associated molecular pattern (PAMP)-like molecule, which can evoke NO generation in macrophages [30], demonstrating a useful model of RAW264.7 macrophages upon LPS stimulation for investigating pathogen-mediated NO signaling cascades. Based on our microarray NO chip analysis and quantitative real-time PCR results, we can conclude that the mechanisms of inhibiting NO release by rSjCa8 may include up-regulated expression of *Catalase-encoding gene* (*Cat*) and down-regulated expression of 47 other genes that include *Myelocytomatosis oncogene* (*Myc*), *Superoxide dismutase 2* (*Sod2*), *Thioredoxin interacting protein* (*Txnip*), *Growth arrest and DNA-damage- inducible 45 alpha* (*Gadd45a*), *TNF receptor superfamily member 6* (*Fas*), *High mobility group box 1* (*Hmgb1*), and *Nitric oxide synthase 2* (*Nos2*), which have been established to be involved in NO production and biological functions [31–40]. Since LPS-stimulated iNOS activation in RAW 264.7 macrophages is reactive oxygen species (ROS)-dependent [41–43], the observation that extremely high expression level of *Cat* (an important molecule in protecting the cell from oxidative damage by ROS) in LPS-pretreated macrophages is induced by rSjCa8 suggests ROS are involved in the inhibitory effect of rSjCa8 on NO release. LPS-stimulated macrophages are a critical component of innate immunity. After LPS treatment, protein-tyrosine kinase mediates phospholipase C phosphorylation, which is followed by increased intracellular calcium concentrations. The increases in calcium levels sequentially trigger the activation of downstream signaling pathways that lead to inducible nitric-oxide synthase [44, 45]. Our findings presented herein clearly show that although rSjCa8 clearly inhibits Toll-like receptor (TLR) activation-induced (e.g., LPS exposure) NO production, non-TLR agonists (e.g., thapsigargin exposure) can abolish the reduction in NO and intracellular Ca^2+^ accumulation in LPS-stimulated macrophages. Thus, inhibition of Ca^2+^-mediated inflammatory pathways likely represents a novel schistosome-induced immunosuppressive mechanism. Deeper insights into the molecules and mechanisms related to the induction of NO production by rSjCa8 both permit a more thorough characterization of the NO signaling system and lead to a better understanding of immune evasion by *S. japonicum*.

Increasing studies have confirmed the existence of cross-talk between Ca^2+^ signaling and NO in response to pathogen invasion [46–48]. To further test our hypothesis that immune evasion of cercariae during skin penetration was mediated by the inhibitory activity of SjCa8 on NO production, we immunized mice with adjuvant + rSjCa8 to induce significantly higher levels of specific anti-rSjCa8 antibodies compared to those in mice immunized with adjuvant or PBS alone (data not shown). Our findings indicate that the inhibitory activity of rSjCa8 on NO production can be suppressed by neutralizing antibodies to rSjCa8. Therefore, accumulation of NO in the challenging skin area of infected hosts caused the partial elimination of invading larvae, which can further illustrate why schistosomal cercariae are scarcely killed or eliminated while penetrating into the host’s skin. Cercariae and skin-stage schistosomula might evade the host immune system-mediated elimination by secreting SjCa8, a stage-specific CBP, which can inhibit macrophage migration and NO release. Therefore, SjCa8 is thought to be a potential chemotherapeutic target or/and vaccine candidate against schistosome infection.

## Conclusion

Our findings support the conclusion that SjCa8, a cercaria and skin-stage schistosomulum specific CBP, inhibits cell migration and Ca^2+^-dependently suppresses NO release by LPS-stimulated RAW264.7 macrophages. It is feasible that, via this mechanism, SjCa8 can contribute to preventing larvae from the damaging or killing effects of macrophages, thus suggesting a further protective effect of this immunoregulatory molecule against host immune attack to promote the invasion, migration, and survival of cercariae. Our findings provide molecular insights that could be harnessed to develop a novel effective vaccine or drug against cercariae invasion for the prevention and control of schisotosomiasis.

## Additional file

Additional file 1:
**Microarray Analysis of 84 Genes Involved in NO Signaling.** (XLSX 20 kb)
